# Mapping novel psychoactive substances policy in the EU: The case of Portugal, the Netherlands, Czech Republic, Poland, the United Kingdom and Sweden

**DOI:** 10.1371/journal.pone.0218011

**Published:** 2019-06-26

**Authors:** Jessica Neicun, Marthe Steenhuizen, Robin van Kessel, Justin C. Yang, Attilio Negri, Katarzyna Czabanowska, Ornella Corazza, Andres Roman-Urrestarazu

**Affiliations:** 1 Care and Public Health Research Centre, Department of International Health, University of Maastricht, Maastricht, The Netherlands; 2 Novel Psychoactive Substance Unit, Centre for Clinical & Health Research Services, University of Hertfordshire, Hatfield, United Kingdom; 3 Faculty of Health, Medicine and Life Sciences, University of Maastricht, Maastricht, The Netherlands; 4 Institute of Public Health, University of Cambridge, Cambridge, United Kingdom; University of Toronto, CANADA

## Abstract

**Introduction:**

The rapid rise in trade and use of NPS and the lack of information concerning their potential toxicity pose serious challenges to public health authorities across the world. Policy measures towards NPS taken so far have a special focus on their legal status, while the implementation of a public health strategy seems to be still missing. The aim of this study is to perform a general assessment of NPS-related policy (including regulatory measures and public health strategies) implemented by six European countries: Portugal, the Netherlands, Czech Republic, Poland, the United Kingdom and Sweden.

**Methods:**

Six EU countries were included in this scoping review study. Drug policies (including legal responses and public health strategies) were analysed. UNODC drug policy classification system was used as a benchmark, while path dependency approach was used for data analysis; a net of inter-dependencies between international, EU and national policies was highlighted.

**Results and discussion:**

The countries included in this study can be placed in a wide spectrum according to their formulation of drug policy, from Portugal and the UK that have specific legal responses to NPS but have differently focused on harm reduction strategies at one end, to Sweden whose drug-free society goal is not translated into a specific regulation of NPS at the other end.

**Conclusion:**

The findings of the study reveal limited development towards harmonisation of national drug policies–particularly with regard to NPS. To tackle the challenge presented by NPS, EU Member states have formulated legislation and public health strategies independently. National approaches to NPS are therefore in line with their already existing drug policies, reflecting cultural values towards substance abuse and national political interests, while the homogenization at an international level has so far mostly been focused on law enforcement and drugs use preventive strategies.

## Introduction

The emergence of novel psychoactive substances (NPS), also misleadingly known as ‘legal highs,’ together with the unprecedented development of an online global drug market, has produced significant challenges for public health and drug policy[1]. NPS consist of a diverse set of substances that have generally been defined as drugs controlled by neither the 1961 Single Convention on Narcotic Drugs nor the 1971 Convention on Psychotropic Substances and tend to either be pharmacological analogues of currently prohibited compounds or newly synthesized chemicals without previous reports of human use[2]. The surge of NPS has changed the face of the drug scene rapidly due to innovative online marketing and commercialization strategies, diffusion of cryptomarkets and, more importantly, the capacity of suppliers and manufacturers of NPS to adapt and circumvent existing drug legislation[3,4]. The rapid rise in trade and use of NPS and the lack of information concerning their potential toxicity pose serious challenges to public health authorities across the world[5]. Authorities have faced a wide range and ever-expanding list of readily available NPS, with lack of chemical and structural consistency among different subgroups and unknown potential harms facing users who try newly-synthesized substances[3,5–7]. Harm linked to NPS has been associated with both the lack of information regarding chemical stability and metabolism, and the unknown addiction potential and serious adverse effects resulting from the unstudied acute and chronic toxicity that NPS might have[5–11].

Since the implementation of the scheduling scheme introduced in 1961 and 1971 by the UN Conventions on drugs, it seems that policy measures have mostly been focused on law enforcement as a response towards substance abuse. Regarding NPS, measures taken so far have a special focus on their legal status, while the implementation of a public health strategy seems to be still missing[12,13]. Therefore, the aim of this study is to perform a general assessment of NPS-related policy (including regulatory measures and public health strategies) implemented by six European countries that have so far adopted heterogeneous national approaches to drug use: Portugal, the Netherlands, Czech Republic, Poland, the United Kingdom and Sweden. Our analysis included countries that: (a) have modified or rejected prohibitionist approaches in their response to drugs, such as Portugal and the Czech Republic, which decriminalised minor drug offenses 16 and 12 years ago, respectively, with no substantial increase in drug use, and the Netherlands, which has a long history of more than 40 years of decriminalisation and tolerance towards drug use; or (b) apply prohibition or supply reduction measures of NPS to preserve public health and safety, such as the UK through the blanket ban introduced in 2016, Poland through its penalisation of any drug possession, and Sweden through its drug-free society goal. By comparing both groups of countries, our objective is to provide a cross-national comparative analysis of the articulation between regulatory measures and public health strategies implemented to tackle NPS use across Europe. This comparison will allow us to assess whether there is a difference regarding the public health focus between countries that apply decriminalisation and countries that have a prohibitionist approach on drugs and NPS. In so doing, this study will be informative for policy makers across Europe.

## Methodology

The cross-national comparative analysis presented is based on a descriptive policy approach, including the collection, organisation and description of legal instruments and formally adopted texts intended to define the course of action in regard to NPS use and public health within the six jurisdictions under study (Portugal, the Netherlands, Czech Republic, Poland, the United Kingdom and Sweden)[14–17].

### 2.1 Data collection and search strategy

This comparative analysis is based on a scoping review that applies a structured qualitative policy analysis of NPS policies at three different layers of action: international (UN), supranational (EU), and national. Our search strategy is based on a scoping review as defined by Arksey & O’Malley[18]. The aim of our scoping review is to comprehensively address broad research questions and to map the available literature in a structured way. Based on our prior knowledge of the subject, the starting point of our search strategy was the collection of the three international legal instruments dealing with drug use (i.e. UN Single Convention on Narcotic Drugs of 1961, UN Convention on Psychotropic Substances of 1971 and UN Convention Against Illicit Traffic in Narcotic Drugs and Psychotropic Substances of 1988), as well as all the legal instruments listed on the NPS section of the European Monitoring Centre for Drugs and Drugs Addiction (EMCDDA) website. Additionally, the country legal profiles and national drug reports prepared by the EMCDDA for the six countries under study were also selected and analysed. Subsequently, a set of nationally relevant documents was identified and retrieved; this selection constituted the core of our data. Finally, a structured search was carried out using the following key terms: NPS, enforcement, drug policy, drug strategy, substance use, prevention, harm reduction, risk minimization, treatment and combinations of these. Data were retrieved from institutional websites as follows: UN: (1) http://www.unodc.org, (2) https://www.un.org/ecosoc; EU: (3) http://www.emcdda.europa.eu, (4) http://www.eur-lex.europa.eu; Portugal: (5) http://sicad.pt; The Netherlands: (6) https://www.rijksoverheid.nl, (7) https://www.trimbos.org; Czech Republic: (8) http://rvkpp.vlada.cz, (9) http://www.drogy-info.cz; Poland: (10) http://www.kbpn.gov.pl, (11) http://www.cinn.gov.pl, (12) https://www.dopalaczeinfo.pl; UK: (13) http://www.legislation.gov.uk, (14) http://www.gov.scot, (15) http://gov.wales, (16) http://www.wales.nhs.uk; Sweden: (17) https://www.government.se, (18) https://www.riksdagen.se. Additional documents were retrieved via snowball sampling[19]. The general inclusion criteria used in our scoping study related to the type of document: 1) legal instruments (laws and regulations); 2) policy documents (national drug strategies and evaluations); 3) national reports on drug-related issues; 4) reports on drug-related issues published by international institutions; 5) drug policy analysis.

### 2.2 Theoretical framework

It is important to consider that drug policy is multidimensional; it might focus on different aspects of drug use and drug-related issues (law enforcement and criminal justice, health, education, social and economic functioning) or intervene at different levels (from global to local) and target different populations (drug users, high-risk groups, general population)[14]. Moreover, drug policies implemented by countries are usually multifocal, simultaneously acting at different levels and realms (i.e. law enforcement, health, education, etc.) while impacting on a variety of target groups. As a result, a wide array of drug policy classification schemes is already available, making the classification task a bit arduous. For the purpose of this paper, the analysis of national drug policies is based in two complementary approaches which reflect their complexity in a suitable, thorough manner.

First, given the broad international harmonisation of drug laws ensuing from the adoption of 1961, 1971 and 1988 UN Conventions that regulate supranational (European Union) and national responses to illicit drugs, the United Nations Office on Drugs and Crime (UNODC) classification system was used as a benchmark. This classification scheme is based on its four major guiding principles for drug control plans: 1) control and reduction of supply; 2) suppression of illicit trafficking; 3) reduction of illicit demand (including prevention, treatment and rehabilitation); 4) cross-sectoral strategies[14]. For the purpose of this paper, measures implemented to reduce drug-related harm and whose goal is not abstinence (so that do not fall under the header of prevention and/or treatment) will be labelled harm reduction.

Second, we used path dependency for our analysis to characterize drug policy harmonisation at a national level, what is to say to analyse the hierarchical processes in which rules and regulations are passed down from an international authority to national governments, with the latter deliberately submitting to international rules, and therefore introducing changes into domestic legal and policy frameworks[20].

### 2.3 Data analysis

According to path-dependent analysis, two dominant types of sequences are present throughout the policy harmonisation process. The first type is a feedback mechanism, modelled as a self-reinforcing positive feedback process[21]. This process entails that when a policy direction is adopted by an international institution such as UNODC or the EU, it states a vision on what is suitable or appropriate to implement in a particular field. It also constitutes an initial precedent for future decisions. As national governments adopt their strategic guidelines in coherence with this given framework, the policy legitimation process is progressively reinforced. Besides, the implementation of policy measures through harmonisation process implies economic and political investment for countries. As a result, the initial steps taken in a particular direction induce further movement in the same way because the relative benefits brought by this pathway progressively increase over time (compared with other possible options), making more difficult to change direction. The moment the choice is made to follow a certain pathway, is referred to as a “critical juncture”[21,22]. The legitimation process is also reinforced by a temporary feedback mechanism according to which events that occur in such sequences are logically ordered and, hence, causally connected. This would entail that a particular legislative proposal is a logical effect of previously chosen polices[21]. Based on this postulate, the analysis of legal instruments and policy documents was guided by a temporary sequence principle whose starting point was the international drug legal framework established by the UN conventions of 1961, 1971 and 1988, and their subsequent transposition into national legal systems. Likewise, EU Action Plan to Combat Drugs (2000–2004) is considered as the starting point in the adoption of drug-related public health strategies. At this level of analysis, the UNODC classification system was applied to analyse the components and main focus of national drug strategies.

Finally, path dependency approach on institutional change gives a central role to national history and traditions, according to which the imposition of a same legal directive results in divergent outcomes. This is particularly pertinent in a policy field like drug use, where different philosophical paradigms guide national political decisions[21,23].

## Results

**Fig 1:**
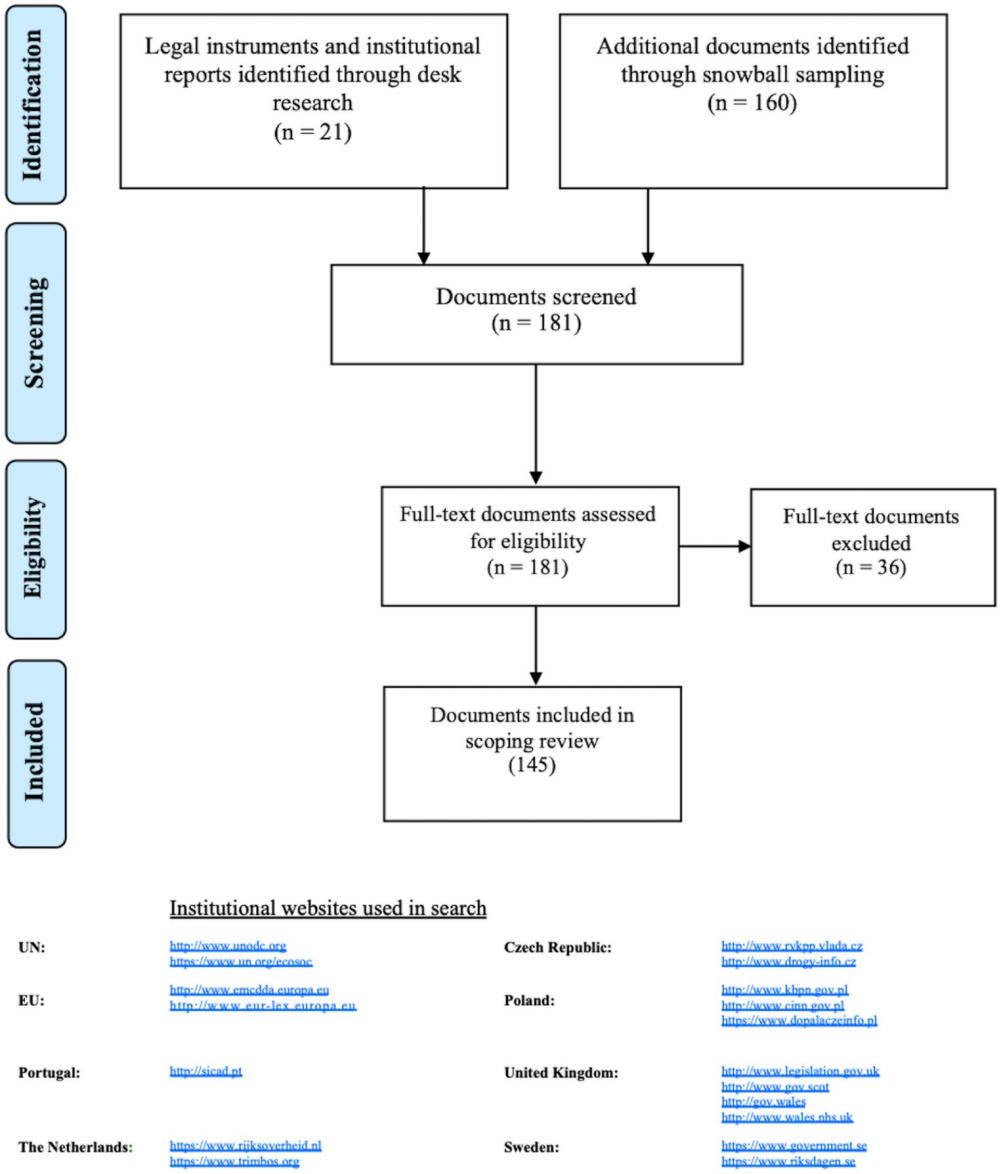
NPS Policy Mapping. Scoping Review Flow Diagram.

The analysis presented is based on the research and analysis of recent legal instruments, formally adopted texts, institutional reports, academic papers and press articles from each of the six jurisdictions under study. Overall, 145 documents were selected and analysed: 52 laws and regulations; 32 policy documents; 27 national reports on drug-related issues; 22 reports on drug-related issues published by international institutions; 9 academic papers; 3 press articles (Fig 1).

The results will be presented narratively, with a sequenced description going through international, supranational and national levels[21]. Subsequently, results will be shown in a comparative table including the main drug policy features of the six jurisdictions under study, classified according to the complementary approaches described above. Finally, in order to better illustrate the national drug policy features, a comprehensive overview of legal measures, national drug strategies and NPS-specific programmes will be provided.

### 3.1. International and supranational drug policy framework: Towards the political balance between law enforcement and public health

The UN Single Convention on Narcotic Drugs of 1961, the UN Convention on Psychotropic Substances of 1971, and the UN Convention Against Illicit Traffic in Narcotic Drugs and Psychotropic Substances of 1988 have framed all subsequent legislation at an international level[24]. In order to control and limit the use of narcotic drugs and psychotropic substances, they are classified according to their therapeutic value, risk of abuse and health dangers, and listed in the Schedules annexed to these UN Conventions. Amendments to the list of substances of the 1961, 1971 and 1988 Conventions are the result of a risk assessment carried out by WHO Expert Committee on Drug Dependence (ECDD), which issues recommendations on changes to the list to the Commission on Narcotic Drugs (CND) based on medical and scientific evaluations. The shortfalls of this approach became clear in 1996, when the first mention of surveillance of non-scheduled substances was expressed at the UN level, and the UN Economic and Social Council (ECOSOC) requested for the establishment of an international special surveillance list of non-scheduled substances which were known to be used in illicit drug trafficking[25]. In turn, the method of scheduling itself was challenged for the first time in 2001, when the rapid pace of changes concerning psychoactive substances and the change in their consumption pointed out the scale of the NPS problem[26,27].

By 2005, the UN formally acknowledged the challenge posed by NPS and officially declared that they may represent threats to public health comparable to those substances that were already scheduled. In its resolution 48/1, UN observed the need for information-sharing concerning the abuse of non-scheduled substances as a new trend. They were also aware of the need of Member states to cooperation[28]. In 2007, the UN urgently repeated the need for Member states to adopt appropriate legislation for the illegal distribution of internationally controlled licit substances via the Internet, due to its serious risk to global health[29]. On year later, the UNODC launched the Global Synthetics Monitoring Analysis Reporting and Trends (SMART) to enhance national responses to the synthetic drug problem[5]. By 2012, the term NPS was mentioned for the first time by the UN in resolution 55/1[30]. Before this, they were generically referred to as non-scheduled substances, although the EU and different Member states already informally used the term NPS. Along with the promotion of measures intended to reduce demand and supply of illegal drugs, the UN expanded the scope of its response to the challenge and called upon Member states to cooperate on the public health response towards NPS for the first time[30]. In 2013, resolution 56/4 stated that the public health response provided by Member states should include tailored prevention strategies to tackle the widespread public understanding of NPS as “safe” substances as they are not subject to traditional drug control instruments. This resolution also exhorted Member states to continue to share information on NPS through its Global SMART Programme[31], and was the basis for the Early Warning Advisory (EWA) that was established in 2013[5]. In 2014, the UN urged the Member states to use the scheduling process of the Conventions of 1961 and 1971[32]. Thus, NPS were considered for scheduling for the first time during the 36^th^ Meeting of the WHO Expert Committee on Drug Dependence in 2014[33]. The most recent document concerning NPS are resolution 58/3, focused on the prevention of the use of NPS by children and young people[34], and resolution 59/8 aimed to address the diversion of non-scheduled precursors[35].

At a supranational EU level, priority was given for the first time to the dangers of synthetic drugs during the meeting of the European Council in Dublin in 1996, which resulted in the expression of need for increasing information, data exchange, risk assessment and control of new synthetic drugs by EU countries[36]. The concerns expressed during this meeting resulted in Joint Action no. 97/396/JHA, which covers the exchange of information towards the European Monitoring Centre for Drugs and Drug Addiction (EMCDDA, created in 1993[37]) through the European Information Network on Drugs and Drug Addiction (REITOX), the establishment of an Early Warning System (EWS), and the possibility of risk assessment by the Commission[38].

The current legal framework in the EU is based upon Council Framework Decision 2004/757/JHA, which outlines minimum provisions on the constituent elements of criminal acts and penalties in the field of illicit drug trafficking. The decision refers to drugs covered by the UN Conventions and, as a result, this framework applies to NPS when they become scheduled[39]. This was followed by Council Decision 2005/387/JHA, in which the information exchange, risk assessment and control of new psychoactive substances were formulated. The joint action establishes the process of risk assessment and the procedures for how NPS can be brought under control by the individual Member states, by order of the EU[40].

The Commission assessed Council Decision 2005/387/JHA in July 2011 and concluded that, although it is a useful instrument, it was insufficient because of the scale and complexity of the problem. On top of that, Regulation (EC) No 1920/2006 of the European Parliament and of the Council of 12 December 2006 on the EMCDDA was published. This regulation mandated the EMCDDA to provide the Community and the Member states with factual, reliable, objective and comparable information at a European level concerning drugs and drug addiction and their consequences. The regulation also stated that every EU Member state needed to designate national institutions or agencies responsible for data collection and reporting on drugs and drug addiction, which are called “national focal points” and became members of REITOX[41].

In 2013, the European Commission released a draft proposal for a regulation on NPS to the European Parliament and Council[42]. This proposal, which would replace 2005 framework decision, aimed to strengthen EU policy by accelerating existing legislative processes through the introduction of an immediate temporary ban (up to 12 months) on substances suspected to present a public health risk. The regulation divided NPS into three different categories: low risk, moderate risk, and severe risk; depending on its risk category, a NPS would flow freely through the internal market, face a temporary ban, or be permanently restricted. It would therefore set a mechanism for information exchange on risk assessment, establishing rules for the movement of NPS in the EU internal market[42,43]. However, this regulation was criticized based on the less prominent role given to the building of evidence-based policy, while the introduction of stricter control measures did not ensure any positive effect on NPS market and harm reduction[43,44]. The most recent document concerning NPS was a proposal written by the European Commission for a regulation of the European Parliament and of the Council amending Regulation (EC) No 1920/2006 in regard to information exchange, early warning systems and risk assessment procedures on NPS[45]. The Council and Parliament accepted this proposal on the 29^th^ of May 2017. The legislation came into force in November 20017 and will become applicable 12 months after that date. It will enable the EU to streamline the procedure for assessing the potential negative effect of a NPS and to implement a ban[46].

Focus on public health was first expressed in 1998, when the European Parliament issued a recommendation for the special session of the UN General Assembly on drugs, in which it expressed that emphasis should be placed on the social aspects of drug problems, a reduction in demand, and a reduction in health risks[47]. This approach was confirmed again in 2000, through the EU Action Plan to Combat Drugs (2000–2004) in which prevention, harm reduction and treatment were included among the main targets. It also stated that more information was required on new synthetic drugs coming to the drugs markets. Later, the European Parliament gave recommendations to the Council and the European Council on the EU Drugs Strategy (2005–2012). The European Parliament wrote that anti-drug policy should be regarded as a form of social intervention and therefore harm reduction strategy should be given priority[48]. In the communication from the European Commission for the EU Drugs Strategy (2005–2012), the problem of psychoactive substances was also included in the field of public health. The plan proposed that drugs should be tackled by a comprehensive approach, which entails that prescription drugs abuse and treatment should also be part of the approach. The document also mentions prevention as an important approach to drugs[49]. The EU Action Plan follows this on Drugs 2005–2008, which advocates for the prevention of licit and illicit psychoactive substances at schools or as widely implemented as possible[50].

In the EU Drugs Action Plan 2009–2012, the need for the spread of evidence-based treatments concerning new drugs was identified as a priority[51]. This was also expressed in the EU Drugs Strategy 2013–2020, in which there is more focus for the specific challenges that NPS represent. In this strategy, the EU prioritized multinational efforts for the development of demand reduction mechanisms including NPS. The role that should be given to new communication technologies and the position they play in facilitating NPS use was also identified as an area of increasing importance. Moreover, there was a growing recognition of the need for EU institutions, bodies and Member states to increase capacity to detect, assess and respond rapidly and effectively to NPS in a direct endorsement of previous work carried out by the EMCDDA. This strategy also prioritized research concerning NPS[48]. These priorities are also expressed in goals in the EU Action Plan 2013–2016. This plan additionally promotes the introduction and adoption of new legislation concerning NPS[42]. The EU Action Plan 2017–2020 elaborates on the points concerning legislation and the response towards new communication techniques. Concerning the strengthening the identification of NPS, the new EU Action Plan includes support for identifying NPS and creating a common methodology for identifying NPS across Member states[52].

Overall, the adoption of UN Conventions on Drugs by UN member states and supranational institutions like the European Union may be considered as the only proper harmonisation process observed thus far in the field of drug policy. Within the EU, what has been observed is the increase in the level of policy convergence through the implementation of policy instruments such as the EU Drug Strategies and Action Plans[53]. EU Drug Strategies have set up the direction and priorities of national drug policies through the definition of general aims, while Action Plans has translated those general aims into specific measurable actions. Nevertheless, in the implementation of such policy instruments the focus has mainly been concentrated on producing and gathering data or research methods rather than any larger policy processes. A milestone in the process of convergence have been the creation of the EMCDDA[53]. Through the creation of the EMCDDA the EU has successfully developed networks of experts able to provide reliable and comparative national data in the field of drugs. As a consequence, academic research and governmental information sources has been improved, which has played a crucial role in building common points of reference and evidence-based public policies across Europe[53,54].

As drug policy involves several policy sectors considered politically sensitive to national governments (such as health, justice and foreign affairs), countries tend to retain the decision-making power, being able to shape the legislative integration process[43,53,54].

Beyond national political resistance EU initiatives may have encountered, it is important to note that the legal instruments used by the EU to regulate in this policy area are not binding, as they give countries flexibility to integrate international rules within their own legal systems. As a result, EU mostly acts as a referee in Member states’ actions through actions or recommendations aiming at reducing drug-related health damage, namely through information and prevention[55].

### 3.3. National legal responses and public health strategies on NPS

#### 3.3.1. Portugal

Since the adoption of the International Opium Convention of 1912, and the subsequent enactment of Law 1687 in 1924, Portugal laid the ground of its drug legal framework which thereafter progressively introduced criminalisation principles[56–58].

Although the notion of decriminalisation of drug use was firstly introduced in 1976, its generalized recognition was finally given by the enactment of Law 30/2000, which entered into force on 1 July 2001. This law maintained the status of illegality for using or possessing any unauthorised drug, but -in an innovative fashion-, shifted its treatment from criminal justice to administrative procedures. It also provided health and social protection for drug users through a set of “dissuasive” measures[57,59]. This was followed by Regulation Decree-Law 130-A/2001 of 23th April that introduced district Commissions in charge of providing treatment and full rehabilitation to non-violent drug use offenders[60,61].

Portugal also has specific legal approaches to NPS, namely Law 13/2012 which modified Decree-Law 15/93 by adding mephedrone to the list of controlled substances[56,62]. That year, Regional Decree 28/2012M updated the legal framework for psychoactive substances and prohibited the sale of ‘legal highs’ (NPS) in Madeira[63,64]. In 2013, via Resolution 5/2013 the Parliament issued recommendations on how to tackle the use of NPS through urgent public health measures[65]. Subsequently, Decree-Law 54/2013 was published by the Government, to provide a legal framework for the prevention and protection against advertisement and commerce of NPS[66]. This included the prohibition of the production, export, advertisement, distribution, sale, or simple dispensing of NPS, as well as a coordinated action led by the General Directorate for Intervention on Addictive Behaviours and Dependencies (SICAD). In 2011, Decree-Law 17/2012 created SICAD to reinforce the planning and monitoring component of programs aimed to reduce the consumption of psychoactive substances [56,60,63]. This legislation was accompanied by Ordinance 154/2013, which updated the list of new psychoactive substances under control[63,67].

The procedure used by Portugal to bring new substances under control (list of individual substances annexed to the main drug law) relies on the standard national procedure for amending any primary legislation. The amendment proposal is usually started by the Ministry of Health, who submit the draft for adoption to the Parliament. Once the amendment proposal approved, the amended law is signed by the President to enter into force. The overall length of the procedure is about 12 months[68].

In 1987, the implementation of the harm reduction programme “Projeto VIDA” was the first step towards the articulation of a more comprehensive and integrated drug policy that already covered demand and supply reduction measures[57,60].

Later, the legal basis for harm reduction measures was outlined by Decree-Law 183/2001, implementing several social and health care structures such as contact and information units, drop-in centres, refuges and shelters, mobile centres for the prevention of infectious diseases, low threshold substitution programmes and syringe exchange schemes[61]. Subsequently, cross-sectoral drugs strategies have been implemented, such as the National Plan Against Drugs and Drug Addiction (2005–2012), and its two successive Action Plans (2005–2008 and 2009–2012) [57,69,70].

The current National Plan for the Reduction of Addictive Behaviours and Dependences (2013–2020) and its two Action Plans (2013–2016 and 2017–2020)[63,71,72], are aimed at reducing the availability of NPS in the market through prevention, dissuasion and dismantling of involved networks attacking both demand and supply issues. Priorities in training and communication were defined as the need for intervention concerning life styles and addictive behaviours related to NPS among young people and adults in different settings of consumption (e.g. universities, prisons, workplaces)[63].

Two intervention projects specifically concerning the prevention of NPS have been implemented in Portugal. The Kosmicare Project, whose first phase ran from 2002 to 2008, encompassed harm reduction and risk minimization–including crisis intervention for psychoactive substance users–on festivals. Its second phase, started in 2010, included collaboration with onsite/offsite health services, as well as an evidence-based intervention model aimed at validating harm reduction methods[73]. From 2006, Piaget Agency for Development (APDES) has launched two innovative projects: Check!n whose main goal was to promote the health and safety among partygoers through information campaigns about NPS, drug testing and sexual practices, and Check!ng (2009–2013) that provided *in situ* drug checking to users in parties and festivals[74,75].

#### 3.3.2. The Netherlands

Dutch drug legislation is mostly based on the 1928 Opium Act which was amended in 1976, when the legal distinction between ‘hard’ (List I) and ‘soft’ (List II) drugs was introduced to the Dutch drug policy framework[76–78].

While the possession of small quantities of drugs for personal use is legally punishable by imprisonment, the use of drug as such does not constitute a criminal offence in the Netherlands. First steps towards decriminalisation were made in 1996, when the Public Prosecution Service set out strict conditions under which cannabis may be sold (no minors, no more than 5g, no nuisance, no advertising, no hard drugs). Hence, cannabis use is not legalised, only tolerated by the authorities[79,80].

Regarding NPS, two options are available for bringing new substances under control, both established in Art. 3a of the Opium Act. Through the standard procedure, a draft Order in Council is presented for approval to both Houses of the States General. The overall procedure usually takes 3–6 months and leads to a permanent control. Through the emergency procedure, lists of new substances are provided by Ministerial Regulation, bringing them under immediate control (within 1 week). If the control decision is not withdrawn within a year, the Ministerial Regulation is followed by an Order in Council that confirms substances’ new legal status[68,81].

The coordination point for assessment and monitoring (CAM) of new drugs was created in 1999, establishing a process in which new drugs undergo a risk assessment before inclusion in the Opium Act[82]. Thus, there are tree procedures of risk assessment: 1) fast assessment for high risk substances (completed within 24 hours); moderate assessment (completed within 10 days) for substances whose risk for public health is not acute; and 3) preventive assessment (which may last for several months)[68].

The basic principles of the Dutch Drug Policy are outlined in the white paper “Drug Policy: Continuity and Change” that was published in 1995[83]. In this paper, the focus on minimizing the health and social consequences related to drug use is acknowledged and expanded as one of the core goals of any future Dutch drug policy initiative. Since then, Dutch drug policy have been further outlined through several issue-specific strategies and policy notes or letters to parliament[80,84–87].

There are different surveillance systems in place. Since 2009, the Monitor Drug Incidents (MDI) collects information on drug-related incidents registered by four medical services to provide an indicative basis for monitoring[88]. Since 2013, the drug information and monitoring system (DIMS), which started in 1992, is also required to monitor and report on NPS. This happens through the New Drug Hotline (*Meldpunt Nieuwe Drugs*, MND)[89]. The DIMS also exchanges information with the National Facility Supporting Dismantling (LFO) that detects the (re)introduction of (new) production processes, (pre)precursors and the production or alteration of NPS[89].

A national prevention campaign was established from 2014–2016, which prioritized young people aged between 16 and 24, with activities predominately in recreational settings[89]. Concerning specific prevention for NPS, very few measures have been developed[90]. In 2016, the Trimbos Institute, a private body officially charged of conducting public policy evaluation, developed a special factsheet on the NPS 4-FA, while NGO Jellinek Prevention produced an even more extensive brochure on this drug available on its website[89,91,92].

#### 3.3.3. Czech Republic

In accordance with UN Conventions of 1961, 1971, and 1988, Czech drug legislation starts in 1962 when possession of unauthorized narcotic drugs was defined as a criminal offence, via the Criminal Code Act No. 140/1961 Coll. and the Criminal Procedure Code Act No. 141/1961 Coll[93,94]. At present, the 2010 Criminal Code (Act No. 40/2009), is the major act covering drug-related offences in the Czech Republic[95].

The control over new substances is established by means of the Addictive Substances Act No. 167/1998 Coll., as amended by the Order of the Government No. 463/2013 Coll., regarding the lists of dependency producing substances, and the Act No. 272/2013 Coll., on drug precursors[96–98]. Czech Republic implements a standard procedure (with lists of individual substances annexed to the main drug law) started by the Ministry of Health, who submit an amendment proposal to other members of the government and to selected public administration bodies. The amendment proposal is then submitted for approval to the government and the two chambers of the Parliament, before its final adoption by President signature. The overall length of the procedure is about one year[68]. This inter-ministerial cooperation in the approach towards drugs was reaffirmed in 2001 with Resolution No. 1177/01, which orders the Ministry of Health and the Ministry of Justice to categorize drugs according to their social and health risks[93].

The establishment of the “Early-Warning System on New Drugs” (EWS), ensuing from EU Council Decision 2005/387/JHA, provided a mechanism for the exchange of information about new psychoactive substances and the assessment of their associated risks[99]. After its creation, the first discussion about scheduling NPS arose in 2009, resulting in the criminal prohibition of 33 new substances in April 2011[100]. Sixty-three additional substances were added to the list of controlled substances in 2017[94].

The main principles of Czech public health strategy on drugs have been defined since the 1900s and lately stipulated in Act No. 379/2005 Coll., on measures to protect against the harms caused by tobacco products, alcohol and other addictive substances, that outlined the types of care an individual who uses addictive substances will receive[99,101,102]. The first and second Government Drug Policy Concept and Programme that covered the period 1993–2000[103] were followed by two consecutive National Drug Strategies (2001–2004 and 2005–2009) and Action Plans. Both the first and second National Drug Strategies (2005–2009) included protection of public health as the main principle of the Czech drug policy, though they did not specifically mention NPS[99,102–105]. The current cross-sectoral National Drug Policy Strategy (2010–2018)[101] does mention that new concerns have arisen concerning the spread of synthetic drugs, but it is not clear whether it encompasses NPS, leaving an important policy gap and public health challenge for the Czech Republic[106].

#### 3.3.4. Poland

In Poland, since the amendment introduced to the 1997 Act on Counteracting Drug Addiction in 2000, any drug possession of is a criminal offence, while the use of drugs itself is not penalised by polish law[107,108]. The current drug legal framework is based on the Act on Countering Drug Addiction of 2005, whose enactment firstly introduced a list of controlled substances annexed to the main drug, as well as preventive and treatment-oriented measures[109].

Consecutive amendments to the 2005 Act have been introduced since 2009. The amended Act of 20 March 2009 introduced control over two new substances (BZP, JWH-18) and 15 plants. In 2010, additional amendments to the Act on Counteracting Drug Addiction and the Act on State Sanitary Inspection were issued[110–112]. Those legal changes also introduced a definition of NPS (or “substitute means”) as a natural or synthetic substance used instead or for the same purpose as a narcotic drug or psychotropic substance, whose manufacture and commercialisation are not regulated under polish law. As a result, mephedrone and a group of synthetic cannabinoids were placed under control[111,113]. In parallel, control over new substances has been delegated to the State Sanitary Inspection, which has therefore the right to withdraw a ‘substitute drug’ for up to 18 months to assess its safety, whenever there is a justified suspicion that it might pose a threat to life or health[111]. In 2011, a new amendment to the Act on Counteracting Drug Addiction was adopted, resulting in the control of 23 new substances[111]. The Working Group for NPS in the Council for Counteracting Drug Addiction was established in 2011 and the Minister of Justice issued a Regulation in 2012 on collecting information on the use of narcotic drugs, psychotropic substances and substitutes[110]. In 2013, an new amendment to the Act on Counteracting Drug Addiction was published; it introduced the risk assessment mechanism of NPS before undertaking control measures[110]. In 2015, another amendment was made to the Act on Counteracting Drug Addiction. This amendment introduced a new definition of NPS including a list, a risk assessment team and increased competences of Custom Services. The amendment also banned 114 NPS[114,115].

The most common way to bring a new substance under control is the standard procedure (individual listing system), triggered by the National Bureau for Drug Prevention (a Ministry of Health’s subordinated body in the field of drug use). This general legislative procedure–which has an average length of 9 months–includes the preparation of a draft law, its subsequent examination by the Council of Ministers and the two Parliamentary chambers, and the final adoption by President signature. In cases of urgency, a rapid amending procedure–whose parliamentary and governmental examination is shorter (6 months)–may also be launched by the Council of Ministers upon proposal of the Ministry of Health[68].

A range of educational campaigns and preventive actions were launched in the early 1990s, introducing the first methadone prescription programme[107]. Later, the first comprehensive National Program for Counteracting Drug Addiction, covering prevention and supply reduction, was implemented in 1999[107,110]. In 2006, national drugs strategies gained a legal status, promoting sustainable approaches to drug use and drug addiction[110]. This was followed by the implementation of the cross-sectoral National Programmes on Counteracting Drug Addiction (2006–2010 and 2011–2016) and the current National Health Programme (2016–2021), whose aim is to reduce drug use and drug-related social and health problems[107,110,115–117].

Prevention concerning NPS started in 2009, with an awareness campaign addressed to young people (“NPS will burn you out. Face the facts”) and the first prevention campaign for NPS that targeted parents and educational communities (“NPS-burn out”)[114]. Between 2013–2015, several prevention campaigns concerning NPS use were launched: i) Universal prevention programme “Taste of life–NPS debate”; ii) Guidebook for parents “Closer to each other–further away from drugs”; iii) Scenario for a 2-lessons parental meeting on NPS to be held at schools; iv) Guidebook for parents “On pharmaceuticals, cannabis and new psychoactive substances without hysteria”[111]. In July 2015, the *Social Pact Against NPS* was signed, aiming to coordinate the activities of public institutions and civil society organisations concerning NPS. At the same time, the social campaign “NPS steal a life”, with the aim to raise awareness among young people about the dangers of NPS started[114].

#### 3.3.5. United Kingdom

In the United Kingdom, the Misuse of Drugs Act 1971 (MDA) is the main law regulating drug control. It divides drugs into three classes, A, B and C[118] and, together with its associated regulations, namely the Misuse of Drugs Regulations 1985[119], provide an extensive provision for the control of dangerous drugs[120]. Under the MDA, it is the possession of the drug–not its use–that constitutes a criminal offence[121]. The Drugs Act 2005 introduced amendments to the MDA and the Police and Criminal Evidence Act 1984, strengthening police powers in relation to drug use[120,122]. In relation to NPS, the UK Government was pressured to act due to the media coverage linked to the death of two teenagers, who presumably died after taking mephedrone in 2010[123]. The first act that effected NPS was thereafter published in 2011, the Police Reform and Social Responsibility Act[124], which facilitated the legislative response to NPS, and introduced a temporary class drug order[121]. Since then, many policy recommendations documents regarding NPS have been issued, advising local authorities on how to act against head shops selling NPS and promoting the development of an evidence-based public health strategy including harm reduction components[125–128]. The UK government has also been trying to increase awareness of NPS-related risks by public health campaigning that was seen as vital to enable potential users to make an informed choice about the drugs they are taking[129,130].

In 2016, the Psychoactive Substances Act was enacted[131] criminalising production, supply or possession with intent to supply of any substance with “psychoactive effects”. The blanket ban replaced the substance-by-substance approach and gave police and other law enforcement agencies greater powers to tackle NPS trade. Under this Act simple possession of NPS does not constitute an offence unless it takes place within a custodial institution. The latter is also considered as an aggravating factor for supply offences, along with the proximity to educational facilities and the use of minors as couriers[121].

In regard to public health, the first drug strategy white paper ‘Tackling Drugs Together’ was launched in 1995 and lasted until 1998[132], when the new ten-year Drug Strategy ‘Tackling Drugs to Build a Better Britain’ was released[133]. This strategy focused on four areas: young people, communities, treatment and availability; it was updated in 2002, but it did not include a specific mention to NPS[134]. Later, the 2010 Drug Strategy aimed at implementing reforms to tackle the problem of emerging NPS from a public health perspective, including the exchange of information on the effects and harms of NPS. It also stated the creation in 2011 of a Forensic Early Warning System (FEWS) to identify new psychoactive substances, as well as the establishment of international agreements aimed at tackling the international drugs trade[135]. In 2017, the United Kingdom’s 2017 Drug Strategy was launched[136]. This cross-sectoral drug strategy addresses illicit drug problems through two overarching aims: to reduce illicit and other harmful drug use and to increase the rates of people recovering from dependency[121]. In parallel, national drug strategies have been implemented within the UK during the last decades: (a) Wales: “Working Together to Reduce Harm: The Substance Misuse Strategy for Wales” (2008–18)[137]; (b) Scotland: “The Road to Recovery: A New Approach to Tackling Scotland’s Drug Problem” (2008–18)[138] and, “Rights, respect and recovery: alcohol and drug treatment strategy” (2018–28)[139]; (c) Northern Ireland: “New Strategic Direction for Alcohol and Drugs Phase 2” (2011–16)[140]. Strongly focused on public health and harm reduction measures, the latter have sometimes conflicted with UK general guidelines, particularly as regards the implementation of drug consumption rooms, which are considered as illegal according to UK drug legislation.

In 2001, the first prevention campaign properly targeting NPS—“Know the Score”—was implemented by Scotland and addressed young people and people who inject NPS[141]. In England, several preventive measures have been taken since the drugs prevention campaign for England, FRANK, launched in 2003; however, such measures did not include information about NPS until 2013[142,143]. In 2016, Mentor UK Alcohol and Drug Education and Prevention Information Service (ADEPIS), launched an awareness campaign intended to provide teachers and general population with information on how to cover NPS in their alcohol and drug education programmes[144]. In 2017, the Report Illicit Drug Reaction (RIDR) was established. This is a UK-wide online pilot system implemented to collect data on adverse reactions related to NPS in order to improve the knowledge of their harmful effects[145].

#### 3.3.6. Sweden

In Sweden, the general drug legal framework is given by the Penal Law on Narcotics enacted in 1968 (SFS 1968:64)[146]. This law defined narcotics drugs as drugs or goods dangerous to people’s health or life. Goods dangerous to health -a Swedish concept that has no direct equivalent internationally- are those with addictive properties or create a state of euphoria, or goods that can easily be converted to products with such properties or effects[147]. It also entailed that the use, unlawful manufacture, acquisition and possession of drugs are criminal offences, and laid down penalties for drug-related crime based on the severity of the offence[148–150]. Over the following decades, drug policy in Sweden became progressively restrictive, raising the standards for control measures and seeking to achieve a drug-free society[149,151]. The prohibitionist approach to drug use was further reinforced in 1993, when imprisonment was introduced into the scale of punishments for drug abuse[149].

In 1999, the Act on Prohibition of Certain Substances which are Dangerous to the Health (SFS 1999:42) was introduced[152]. This act prohibited certain products that entail a danger to human health or life and may be used with the aim of inducing intoxication or other effects[147]. Later that year, the Government has given the capacity to classify new substances through a list annexed to the Ordinance regarding the Prohibition of Certain Goods Dangerous to Health (SFS 1999:58)[153]. In 2011, the Act on the Destruction of Certain Substances of Abuse Dangerous to Health (SFS 2011:111) was published[154]. This law aims to prevent the distribution of uncontrolled substances, while reinforcing police and customs power to confiscate and destroy psychoactive substances before their official classification as narcotics or dangerous to health[147,150,155]. The Public Health Agency of Sweden, established in 2014, is the authority in charge of monitoring and investigating the need for control of such goods, that are not medical products[147].

The procedure for classifying new substances is started under the recommendation of either the National Institute of Public Health or the Medical Products Agency, which are also responsible for the risk assessment. The standard procedure–that may last 5–6 months–is led by the Government, which introduces amendments to the relevant ordinance. The latter may also introduce rapid amendments to control substances presenting a significant risk of death or widespread abuse[68].

In the realm of public health, successive strategies have been implemented since the publication of the first three-year National Action Plan on Drugs in 2002[156]. This cross-sectoral strategy was followed by the 2004 Swedish Anti-Drug Strategy (ANDT), the Action Plan on Drugs of 2005, and the New Action Plan on Drugs 2011–2015 whose aim was to stress cooperation between the spheres of health promotion, disease prevention, crime fighting, treatment and rehabilitation[147,149,157,158].

In 2016, the Comprehensive Strategy for Alcohol, Narcotics, Doping and Tobacco (ANDT) covering the period 2016–20 was launched[159]. The ANDT strategy is part of the national public health policy and seeks for society free from narcotics and doping, while reducing medical and social drug-related harm[150]. It defines NPS as substances that are likely to endanger human life or health and are expected to meet the criteria for illegal drugs, though they are not listed yet. It propose methods to streamline the process of classification and analyse methods to classify drugs or health hazardous goods into groups and ban them as a single group, instead of separately[150].

Two other initiatives are also relevant to prevention and harm reduction policy. The first is the Network for the Current Situation of Drugs in Sweden (NADiS), which is the Swedish early warning system for NPS[160]. The second is a booklet that was published by the Swedish Agency for Public Health in 2014 that explains what cannabis and synthetic cannabinoids are[161].

## Discussion

In the field of drug policy, the compliance to UN Conventions may be considered as a step towards policy harmonisation based on the adoption of the law enforcement goals (e.g. supply and demand reduction, fight against drugs trafficking), while the focus on public health initiatives–that incorporate harm reduction and rehabilitation services–has been incorporated through the development of European approaches on drug use. In fact, the guiding principles for drug policy development have been defined by international institutions such as the UNODC and have subsequently been adopted by the EU and national governments. According to these general principles, summarised in the UNODC scheme used as a reference in this paper, drug policy should primarily focus on control and reduction of drug supply (including suppression of illicit trafficking), while health-related actions are only considered as ways to reducing demand for drugs. Interestingly, national drug policies are often presented as means to a public health strategy guided by the harm reduction principle, though this does not always seem to be the case of NPS policy as it emerged from our analysis (Table 1).

**Table 1:** Overview of NPS regulatory models, current national drug strategies and NPS-specific programmes.

	Portugal	The Netherlands	Czech Republic	Poland	UK	Sweden
Regulatory model	Decriminalization	Decriminalization	Decriminalization	Prohibitionist	Prohibitionist	Prohibitionist
NPS-specific regulation	Law 13/2012 Decree-Law 15/93 Regional Decree 28/2012/M Parliament Resolution 5/2013 Decree-Law 54/2013 Ordinance 154/2013	NPS are regulated through amendments to relevant schedules of the 1928 Opium Act (Opiumwet), namely: 2002 Opium Act Decision (Wijziging van de Opiumwet) 2011 Opium Act Directive (Aanwijzing Opiumwet Stc 2011–11134)	NPS are regulated through amendments to the Addictive Substances Act No. 167/1998 Coll. via: Act No. 272/2013 Coll., on drug precursors; Order of the Government No. 463/2013 Coll., regarding the lists of dependency producing substances.	NPS are regulated through amendments to: (i) the Act of 29 July 2005 on Counteracting Drug Addiction, as amended in 2009, 2010, 2011, 2013 and 2015 (ii) the Act of 14 March 1985 on State Sanitary Inspection, as amended in 2010	Psychoactive Substances Act 2016	NPS are controlled through amendments to: Penal Law on Narcotics (SFS 1968:64) Act on the Prohibition of Certain Goods Dangerous to Health (SFS 1999:42) Act on the Destruction of Certain Substances of Abuse Dangerous to Health (SFS 2011:111) Ordinance regarding the Prohibition of Certain Goods Dangerous to Health (SFS 1999:58)
Drugs Regulatory body	(i) Council for Drugs, Drug Addiction and Alcohol-Related Problems (inter-ministerial); (ii) General-Directorate for Intervention on Addictive Behaviours and Dependencies (SICAD/Ministry of Health); (iii) Portuguese Economy and Food Safety Authority (enforcement).	Inter-ministerial (Ministry of Health, Welfare and Sport, Ministry of Justice and Security, Ministry of Foreign Affairs)	Government Council for Drug Policy Coordination (GCDPC/Inter-ministerial)	(i) Council for Counteracting Drug Addiction (inter-ministerial); (ii) National Bureau for Drug Prevention (Ministry of Health); (iii) State Sanitary Inspector; customs (enforcement).	Home Office (UK)	Public Health Agency of Sweden
Drugs/NPS Monitoring System	Warning and Denunciation Online System (NPS)	Monitor Drug Incidents (MDI) Drug Information and Monitoring System (DIMS) New Drug Hotline (MND) National Facility Supporting Dismantling (LFO)	National Monitoring Centre for Drugs and Addiction	NPS are listed by the Ministry of Health	Forensic Early Warning System (FEWS) inactive since the blanket ban introduced by the 2016 Psychoactive Substances Act.	Network for the Current Situation of Drugs in Sweden / NADiS and NADiS-portal (NPS)
NPS control procedure	Standard procedure (up to 12 months): individual list of substances annexed to the main drug law (Decree-Law 15/93)	Standard (3–6 months) and emergency procedure (1 week); individual list of substances annexed to the main drug law (1928 Opium Act).	Standard procedure (up to 12 months): individual list of substances annexed to the main drug law (Order of the Government No. 463/2013 Coll.)	Standard (up to 9 months) and rapid procedure (up to 6 months); individual list of substances annexed to the main drug law (Act on Counteracting Drug Addiction of 2005).	Blanket ban	Standard procedure (5–6 months); individual list of substances annexed to the revelant Ordinance (SFS 1999:58)
Current National Drug Strategy	National Plan for the Reduction of Addictive Behaviours and Dependence 2013–20, and its Action Plans 2013–16 & 2017–20	Policy view on drug prevention addressing youth and nightlife (2015)	National Drug Policy Strategy 2010–18	National Health Programme (2016–21), supported by 3 additional strategies: (i) National Programme for Resolving and Preventing Alcohol-Related Problems; (ii) National Programme for Combatting Health Consequences of Using Tobacco and Related Products; (iii) Behavioural Addictions Strategy.	(a) Drug Strategy 2017 (UK) (b) Working Together to Reduce Harm: The Substance Misuse Strategy for Wales 2008–18 (Wales) (c) 2018–28 Rights, respect and recovery: alcohol and drug treatment strategy (Scotland) (d) New Strategic Direction for Alcohol and Drugs Phase 2: 2011–16 (Northern Ireland)	Comprehensive Strategy for Alcohol, Narcotics, Doping and Tobacco (ANDT) 2016–20
National Drug Strategy’s focus	Cross-sectoral strategy: Supply and demand reduction	Cross-sectoral strategy: (i) control and reduction of supply; (ii) suppression of illicit trafficking; (iii) reduction of illicit demand (prevention, treatment and rehabilitation).	Cross-sectoral strategy: (i) prevention; (ii) treatment and reintegration; (iii) harm reduction; (iv) supply reduction.	Cross-sectoral strategy: (i) prevention; (ii) treatment, rehabilitation, harm reduction and social reintegration; (iii) supply reduction; (iv) international cooperation; and (v) research and monitoring.	(a) UK: Cross-sectoral strategy: (i) reducing demand; (ii) restricting supply; (iii) building recovery; (iv) global action. (b) Wales, (c) Scotland & (d) Northern Ireland: Cross-sectoral: (i) Harm reduction; (ii) community protection; (iii) control of supply.	Cross-sectoral strategy: (i) prevention; (ii) harm reduction.
NPS-specific programmes						
Programme name	(i) Kosmicare project (since 2002)	(i) Factsheet 4-FA (2016)	N/A	(i) Taste of life—NPS debate (2013)	(i) Know the Score (Scotland, 2001)	(i) Cannabis—let facts guide your decisions (2014)
*Intervention paradigm*	*Harm reduction and risk minimization*	*Prevention (outreach campaign)*	N/A	*Prevention (outreach campaign)*	*Prevention (outreach campaign)*	*Prevention (outreach campaign)*
*Target population*	*Young people and adults (recreational settings)*	*High risk groups and young people*	N/A	*General population*	*General population*	*General population*
Programme name	(ii) Check!n Project (since 2006)	(ii) Jellinek. 4-FA / 4-FMP. Informatie over alcohol & drugs (2016)	N/A	(ii) Guidebooks for parents (2013–15): "Closer to each other—further away from drugs"; "On pharmaceuticals, cannabis and NPS without hysteria"; Scenario for a 2-lessons parental meeting on NPS	(ii) FRANK (UK, 2013)	N/A
*Intervention paradigm*	*Prevention (outreach campaign)*	*Prevention (outreach campaign)*	N/A	*Prevention (outreach campaign)*	*Prevention (outreach campaign)*	N/A
*Target population*	*Young people and adults (recreational settings)*	*High risk groups and young people*	N/A	*School settings*	*General population*	N/A
Programme name	(iii) Check!ng Project (2009–2013)	N/A	N/A	(iii) Social Pact Against NPS (2015)	(iii) Psychoactive Substances. What schools need to know about the new law (UK, 2016)	N/A
*Intervention paradigm*	*Harm reduction and risk minimization*	N/A	N/A	*Prevention (outreach campaign)*	*Prevention (outreach campaign)*	N/A
*Target population*	*Young people and adults (recreational settings)*	N/A	N/A	*Public and private institutions dealing with the problem of NPS use*	*School settings and general population*	N/A
Programme name	N/A	N/A	N/A	(iv) NPS steal a life (2015)	(iv) Report Illicit Drug Reaction (UK, 2017)	N/A
*Intervention paradigm*	N/A	N/A	N/A	*Prevention (outreach campaign)*	*Harm reduction and risk minimization*	N/A
*Target population*	N/A	N/A	N/A	*Young people*, *parents*, *teachers and others in contact with young people*	*General population*	N/A

**Fig 2:**
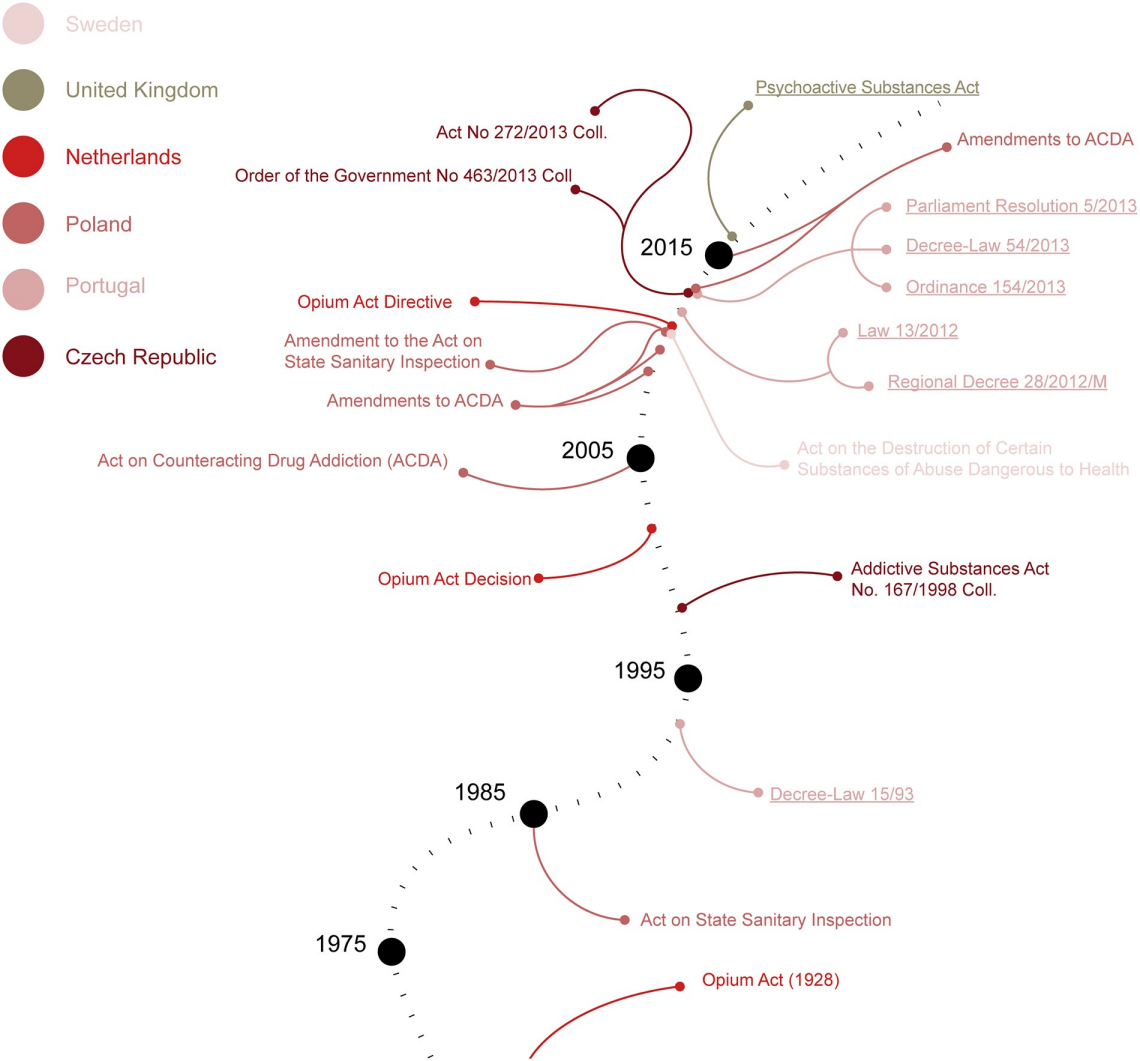
A chronological overview of the legislation that pertains to Novel Psychoactive Substances in the jurisdictions under study. Legislation that is specifically designed for NPS is underlined.

**Fig 3:**
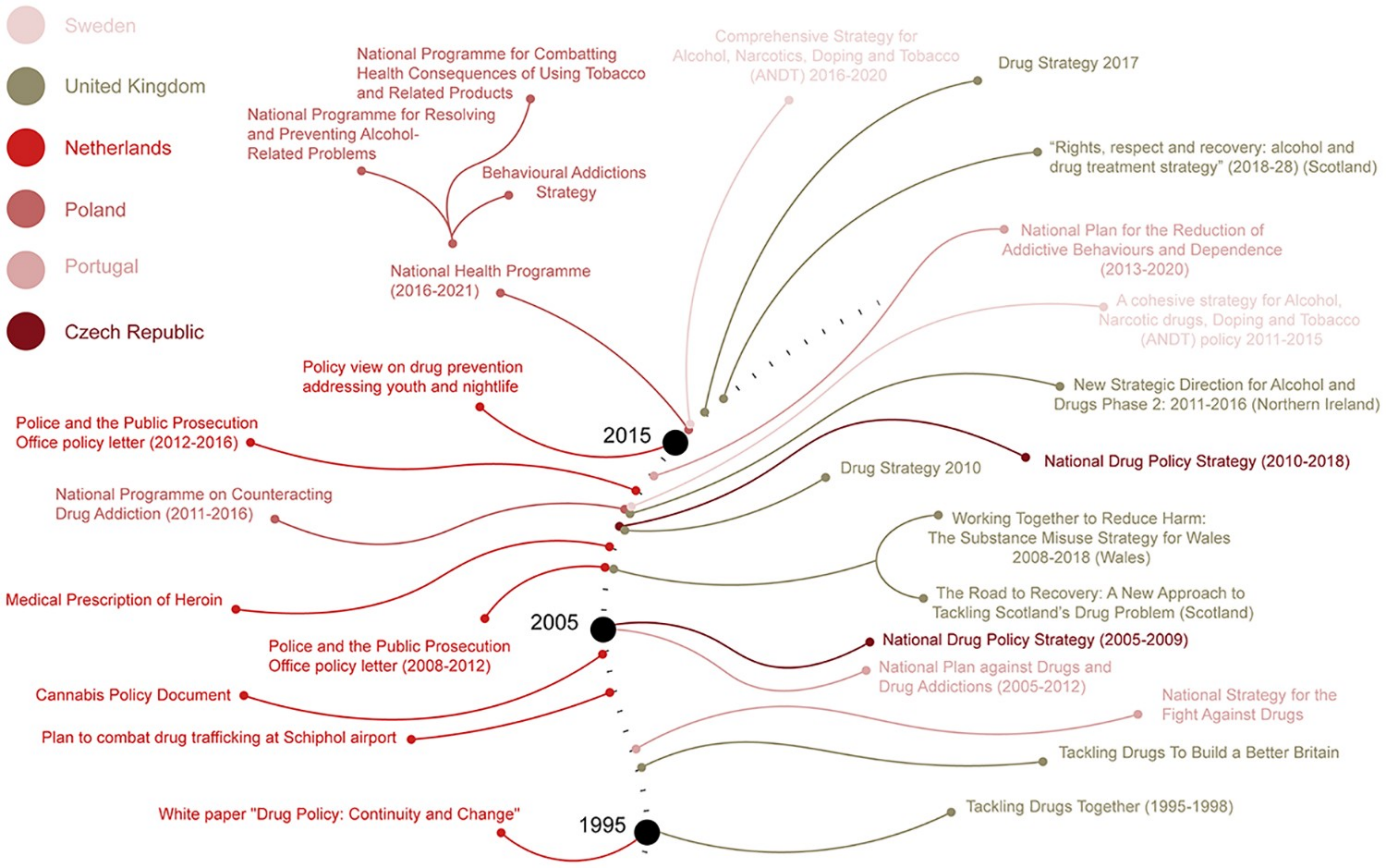
An overview of the national drug strategies adopted by the jurisdictions under study.

**Fig 4:**
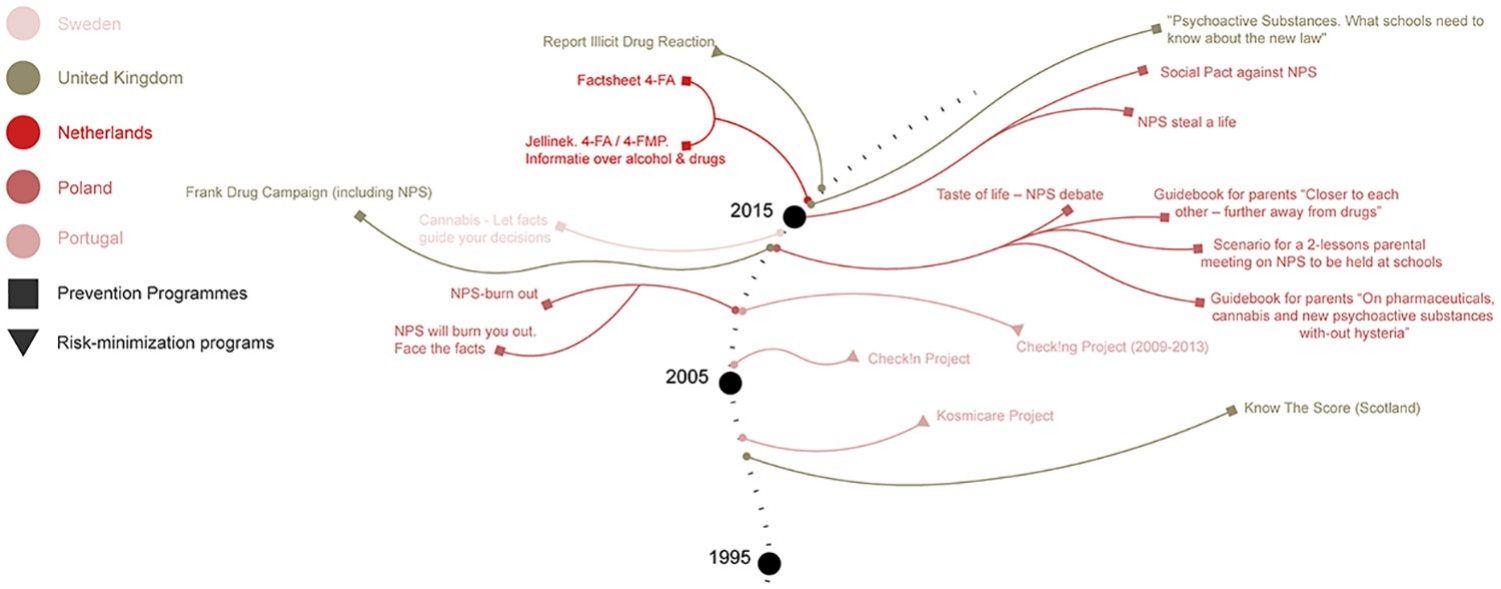
An overview of the NPS-specific programmes implemented by the jurisdictions under study.

The countries included in this study can be placed in a wide spectrum according to their formulation of drug policy, from Portugal and the UK that have specific legal responses to NPS but have differently focused on harm reduction strategies at one end, to Sweden whose drug-free society goal is not translated into a specific regulation of NPS at the other end (see Fig 2). The other EU Member states included in this study may be placed in different points on a continuum, with Poland standing out as the most proliferous in the field of NPS use prevention. In accordance with EU Drugs Strategies implemented since 2000, national drugs policies in the six countries under study may be classified as cross-sectoral as they combine prevention, reduction of drug supply and demand, alongside fight against illicit drug trafficking (see Fig 3). With respect to NPS-specific programmes implemented by most of the countries under study (except for the Czech Republic), it appears that they have focused on the prevention of NPS use, whether through improved information exchange about their chemical composition and hazards or through awareness campaigns on the health risk associated to their use (see Fig 4).

Although the EU has recognized the importance of harm reduction since 2003, it seems that its policy efforts have been focused on the implementation of approaches that tackle different stages of the drug problem, namely the reduction of demand and supply, yet it has not decided whether a more liberal or more repressive drug policy is the better option[162].

In spite of differences in criminal justice systems and policy responses implemented by the countries under study, convergence among them has been observed at the level of policy paradigms through the progressive adoption of some postulates from the harm reduction approach. It has resulted in the widely adopted distinction of drugs by the level of harm they produce, the focalisation of policy response on more harmful drugs and the increasing preference for health treatment instead of criminal justice responses[53]. The adoption of the harm reduction postulates has also resulted in national drug strategies that differently operationalise the goal of reducing health damages produced by drug use. With regard to NPS, harm reduction has commonly been translated into prevention campaigns aimed at raising public awareness of the risk carried by NPS use. Portugal and the UK are the only countries having implemented proper risk minimization campaigns (drug testing, crisis intervention for NPS users, online data collection on adverse reactions related to NPS). It is worth to note that the scope of this study only included six European countries, therefore its results cannot be generalized, as more countries should be analysed to get a better picture of the situation across the EU. More research should also be conducted to establish whether strategies towards NPS that are in place have an effect on both the prevention of NPS use and the minimization of the risk associated with their use. Nevertheless, this selection of countries provides a wide overview of the different legal responses and public health strategies adopted in the EU until now, and it may therefore be considered as a benchmark for policy-making process.

Finally, it is important to consider that, since national drug policy and public health strategies are formulated by each Member states based on cultural backgrounds and political priorities, with the EU playing a supranational referee role, harmonisation is not an achievable aim in the current framework[53,54]. In fact, throughout the convergence process countries have adopted strategic guidelines based on the opportunities given by their cultural-bounded approaches on drugs and their particular institutional arrangements, as well as on the assessment of the benefits they would obtain by implementing such legal directives, especially when the latter entails institutional change or political swap. Furthermore, the combined action of the UN and the EU in the field of drug policy has imposed the search of political consensus at international level, reflected in the wide adoption of approaches primarily focalised on supply and demand-reduction.

However, the definition of a specific integrated EU policy towards NPS has emerged as a crucial issue due to both the constant and rapid evolution of this phenomenon and its health-related risks, including mental health damage and mortality. Therefore, we consider that NPS use is an area where the EU should be more proactive in promoting the implementation of risk minimization measures. From the point of view of our study, if the EU wishes to handle the problem of NPS at a European level in the framework of harm reduction, there appear to be two options. The first is to continue to formulate a drug policy that encompasses the large spectrum of national approaches to drug use existing in the EU, which seems to be a less arduous political task considering the huge differences between countries. Following this path EU drug policy would remain focused on information exchange and law enforcement, while its effectiveness in terms of drug use prevention will still need to be proved. The second option for the EU may be to formulate a drug policy that gives priority to harm reduction measures, and that compels Member states to implement them.

## Conclusion

NPS pose an unprecedented threat to public health and a huge challenge to drug policy, due to the unknown short- and long-term health effects and the rapidly evolving market that bypasses current scheduling legislation. The findings of the study reveal limited development towards harmonisation of national drug policies–particularly with regard to NPS. In the context of the ambiguous position held by the EU in adopting and promoting harm reduction as a prior goal of drug policy, there has been observed a predominance of national approaches to drug use. To tackle the challenge presented by NPS, EU Member states have formulated legislation and public health strategies independently. As a result, national approaches to NPS are in line with their already existing drug policies, reflecting cultural values towards substance abuse and national political interests, while the homogenization at an international level has so far mostly been focused on law enforcement and drug use preventive strategies. The lack of an integrated EU drug policy may also be explained by the EU’s need to find compromises between members’ different interests. However, implementing a drug policy which encompass a public health response towards NPS focusing on harm reduction is increasingly important. Although, the EU has not yet demonstrated that it is able to take a strong leadership position concerning drug policy not to say public health harmonisation.

## Supporting information

S1 Table Prisma NPS Policy Mapping checklist. (DOC)
