# Supplementary material for: Mapping novel psychoactive substances policy in the EU: The case of Portugal, the Netherlands, Czech Republic, Poland, the United Kingdom and Sweden
Source: PLoS One. 2019 Jun 26;14(6):e0218011. doi: 10.1371/journal.pone.0218011 (PMC6594604; doi:10.1371/journal.pone.0218011)
Supplement: S1 Table — (DOC) [file pone.0218011.s001.doc]

| **Section/topic** | **#** | **Checklist item** | **Reported on page #** |
| --- | --- | --- | --- |
| **TITLE** | | |  |
| Title | 1 | Identify the report as a systematic review, meta-analysis, or both. | **Page 1:** Mapping Novel Psychoactive Substances Policy in the EU: the case of Portugal, the Netherlands, Czech Republic, Poland, the United Kingdom and Sweden (page1).  This is a scoping review. |
| **ABSTRACT** | | |  |
| Structured summary | 2 | Provide a structured summary including, as applicable: background; objectives; data sources; study eligibility criteria, participants, and interventions; study appraisal and synthesis methods; results; limitations; conclusions and implications of key findings; systematic review registration number. | **Introduction:** The rapid rise in trade and use of NPS and the lack of information concerning their potential toxicity pose serious challenges to public health authorities across the world. Policy measures towards NPS taken so far have a special focus on their legal status, while the implementation of a public health strategy seems to be still missing. The aim of this study is to perform a general assessment of NPS-related policy (including regulatory measures and public health strategies) implemented by six European countries: Portugal, the Netherlands, Czech Republic, Poland, the United Kingdom and Sweden.  **Methods:** Six EU countries were included in this scoping review study. Drug policies (including legal responses and public health strategies) were analysed. UNODC drug policy classification system was used as a benchmark, while path dependency approach was used for data analysis; a net of inter-dependencies between international, EU and national policies was highlighted.  **Results and discussion:** The countries included in this study can be placed in a wide spectrum according to their formulation of drug policy, from Portugal and the UK that have specific legal responses to NPS but have differently focused on harm reduction strategies at one end, to Sweden whose drug-free society goal is not translated into a specific regulation of NPS at the other end.  **Conclusion:** The findings of the study reveal limited development towards harmonisation of national drug policies– particularly with regard to NPS. To tackle the challenge presented by NPS, EU Member states have formulated legislation and public health strategies independently. National approaches to NPS are therefore in line with their already existing drug policies, reflecting cultural values towards substance abuse and national political interests, while the homogenization at an international level has so far mostly been focused on law enforcement and drugs use preventive strategies. |
| **INTRODUCTION** | | |  |
| Rationale | 3 | Describe the rationale for the review in the context of what is already known. | **Page 4-5:** “Since the implementation of the scheduling scheme introduced in 1961 and 1971 by the UN Conventions on drugs, it seems that policy measures have mostly been focused on law enforcement as a response towards substance abuse. Regarding NPS, measures taken so far have a special focus on their legal status, while the implementation of a public health strategy seems to be still missing. Therefore, the aim of this study is to perform a general assessment of NPS-related policy (including regulatory measures and public health strategies) implemented by six European countries that have so far adopted heterogeneous national approaches to drug use: Portugal, the Netherlands, Czech Republic, Poland, the United Kingdom and Sweden.” |
| Objectives | 4 | Provide an explicit statement of questions being addressed with reference to participants, interventions, comparisons, outcomes, and study design (PICOS). | **Page 5:** “By comparing both groups of countries, our objective is to provide a cross-national comparative analysis of the articulation between regulatory measures and public health strategies implemented to tackle NPS use across Europe. This comparison will allow us to assess whether there is a difference regarding the public health focus between countries that apply decriminalisation and countries that have a prohibitionist approach on drugs and NPS.” |
| **METHODS** | | |  |
| Protocol and registration | 5 | Indicate if a review protocol exists, if and where it can be accessed (e.g., Web address), and, if available, provide registration information including registration number. | N/A. There is no protocol since this is a scoping review. |
| Eligibility criteria | 6 | Specify study characteristics (e.g., PICOS, length of follow-up) and report characteristics (e.g., years considered, language, publication status) used as criteria for eligibility, giving rationale. | **Page 5:** “The cross-national comparative analysis presented is based on a descriptive policy approach, including the collection, organisation and description of legal instruments and formally adopted texts intended to define the course of action in regard to NPS use and public health within the six jurisdictions under study (Portugal, the Netherlands, Czech Republic, Poland, the United Kingdom and Sweden). This comparative analysis is based on a scoping review that applies a structured qualitative policy analysis of NPS policies at three different layers of action: international (UN), supranational (EU), and national was used. Our search strategy is based on a scoping review as defined by Arksey & O’Malley” |
| Information sources | 7 | Describe all information sources (e.g., databases with dates of coverage, contact with study authors to identify additional studies) in the search and date last searched. | **Page 6:** “Based on our prior knowledge of the subject, the starting point of our search strategy was the collection of the three international legal instruments dealing with drug use (i.e. UN Single Convention on Narcotic Drugs of 1961, UN Convention on Psychotropic Substances of 1971 and UN Convention Against Illicit Traffic in Narcotic Drugs and Psychotropic Substances of 1988), as well as all the legal instruments listed on the NPS section of the European Monitoring Centre for Drugs and Drugs Addiction (EMCDDA) website. Additionally, the country legal profiles and national drug reports prepared by the EMCDDA for the six countries under study were also selected and analysed. Subsequently, a set of nationally relevant documents was identified and retrieved; this selection constituted the core of our data. Finally, a structured search was carried out using the following key terms: NPS, enforcement, drug policy, drug strategy, substance use, prevention, harm reduction, treatment and combinations of these.” |
| Search | 8 | Present full electronic search strategy for at least one database, including any limits used, such that it could be repeated. | **Page 6-7:** “Data were retrieved from institutional websites as follows: UN: (1) http://www.unodc.org, (2) https://www.un.org/ecosoc; EU: (3) http://www.emcdda.europa.eu, (4) http://www.eur-lex.europa.eu; Portugal: (5) http://sicad.pt; The Netherlands: (6) https://www.rijksoverheid.nl, (7) https://www.trimbos.org; Czech Republic: (8) http://www.rvkpp.vlada.cz, (9) http://www.drogy-info.cz; Poland: (10) http://www.kbpn.gov.pl , (11) http://www.cinn.gov.pl, (12) https://www.dopalaczeinfo.pl; UK: (13) http://www.legislation.gov.uk, (14) http://www.gov.scot, (15) http://gov.wales, (16) http://www.wales.nhs.uk; Sweden: (17) https://www.government.se, (18) https://www.riksdagen.se. Additional documents were retrieved via snowball sampling.” |
| Study selection | 9 | State the process for selecting studies (i.e., screening, eligibility, included in systematic review, and, if applicable, included in the meta-analysis). | **Page 7:** “The general inclusion criteria used in our scoping study related to the type of document: 1) legal instruments (laws and regulations); 2) policy documents (national drug strategies and evaluations); 3) national reports on drug-related issues; 4) reports on drug-related issues published by international institutions; 5) drug policy analysis. “ |
| Data collection process | 10 | Describe method of data extraction from reports (e.g., piloted forms, independently, in duplicate) and any processes for obtaining and confirming data from investigators. | **Page 8-9:** “Based on this postulate, data extraction from legal instruments and policy documents was guided by a temporary sequence principle whose starting point was the international drug law framework established by the UN conventions of 1961, 1971 and 1988, and their subsequent transposition into national legal systems. Likewise, EU Action Plan to Combat Drugs (2000-2004) is considered as the starting point in the adoption of public health strategies encompassing a harm reduction component.” |
| Data items | 11 | List and define all variables for which data were sought (e.g., PICOS, funding sources) and any assumptions and simplifications made. | **Page 6:** “Finally, a structured search was carried out using the following key terms: NPS, enforcement, drug policy, drug strategy, substance use, prevention, harm reduction, risk minimization, treatment and combinations of these.” |
| Risk of bias in individual studies | 12 | Describe methods used for assessing risk of bias of individual studies (including specification of whether this was done at the study or outcome level), and how this information is to be used in any data synthesis. | N/A. There is no bias since this is a scoping review |
| Summary measures | 13 | State the principal summary measures (e.g., risk ratio, difference in means). | N/A |
| Synthesis of results | 14 | Describe the methods of handling data and combining results of studies, if done, including measures of consistency (e.g., I^2^) for each meta-analysis. | N/A. No meta-analysis was conducted. |

Page 1 of 2

| **Section/topic** | **#** | **Checklist item** | **Reported on page #** |
| --- | --- | --- | --- |
| Risk of bias across studies | 15 | Specify any assessment of risk of bias that may affect the cumulative evidence (e.g., publication bias, selective reporting within studies). | **Page 4-5.** “Therefore, the aim of this study is to perform a general assessment of NPS-related policy (including regulatory measures and public health strategies) implemented by six European countries that have so far adopted heterogeneous national approaches to drug use: Portugal, the Netherlands, Czech Republic, Poland, the United Kingdom and Sweden. Our analysis included countries that: (a) have modified or rejected prohibitionist approaches in their response to drugs, such as Portugal and the Czech Republic, which decriminalised minor drug offenses 16 and 12 years ago, respectively, with no substantial increase in drug use, and the Netherlands, which has a long history of more than 40 years of decriminalisation and tolerance towards drug use; or (b) apply prohibition or supply reduction measures of NPS to preserve public health and safety, such as the UK through the blanket ban introduced in 2016, Poland through its penalisation of any drug possession, and Sweden through its drug-free society goal.” |
| Additional analyses | 16 | Describe methods of additional analyses (e.g., sensitivity or subgroup analyses, meta-regression), if done, indicating which were pre-specified. | N/A |
| **RESULTS** | | |  |
| Study selection | 17 | Give numbers of studies screened, assessed for eligibility, and included in the review, with reasons for exclusions at each stage, ideally with a flow diagram. | **Page 9.** Overall, 145 documents were retrieved and analysed: 52 laws and regulations; 32 policy documents; 27 national reports on drug-related issues; 22 reports on drug-related issues published by international institutions; 9 academic papers; 3 press articles. |
| Study characteristics | 18 | For each study, present characteristics for which data were extracted (e.g., study size, PICOS, follow-up period) and provide the citations. | **Pages 5.** This was a scoping review therefore no systematic review was performed. Data was extracted for international/national legal and policy frameworks from the following sources:   1. United Nations:   (1) [http://www.unodc.org](http://www.unodc.org/)  (2) <https://www.un.org/ecosoc>   1. European Union:   (3) [http://www.emcdda.europa.eu](http://www.emcdda.europa.eu/)  (4) [http://www.eur-lex.europa.eu](http://www.eur-lex.europa.eu/)   1. Portugal:   (5) [http://sicad.pt](http://sicad.pt/)   1. The Netherlands:   (6) [https://www.rijksoverheid.nl](https://www.rijksoverheid.nl/)  (7) https://www.trimbos.org   1. Czech Republic:   (8) [http://www.rvkpp.vlada.cz](http://www.rvkpp.vlada.cz/)  (9) [http://www.drogy-info.cz](http://www.drogy-info.cz/)   1. Poland:   (10) http://www.kbpn.gov.pl  (11) [http://www.cinn.gov.pl](http://www.cinn.gov.pl/)  (12) [https://www.dopalaczeinfo.pl](https://www.dopalaczeinfo.pl/)   1. United Kingdom:   (13) [http://www.legislation.gov.uk](http://www.legislation.gov.uk/)  (14) [http://www.gov.scot](http://www.gov.scot/)  (15) [http://gov.wales](http://gov.wales/)  (16) http://www.wales.nhs.uk   1. Sweden:   (17) [https://www.government.se](https://www.government.se/)  (18) [https://www.riksdagen.se](https://www.riksdagen.se/) |
| Risk of bias within studies | 19 | Present data on risk of bias of each study and, if available, any outcome level assessment (see item 12). | N/A The paper only used legal and policy documents therefore no outcome level assessment was performed. |
| Results of individual studies | 20 | For all outcomes considered (benefits or harms), present, for each study: (a) simple summary data for each intervention group (b) effect estimates and confidence intervals, ideally with a forest plot. | N/A The paper only used legal and policy documents therefore no outcome level assessment was performed. |
| Synthesis of results | 21 | Present results of each meta-analysis done, including confidence intervals and measures of consistency. | N/A The paper only used legal and policy documents therefore no outcome level assessment was performed. |
| Risk of bias across studies | 22 | Present results of any assessment of risk of bias across studies (see Item 15). | N/A The paper only used legal and policy documents therefore no outcome level assessment was performed. |
| Additional analysis | 23 | Give results of additional analyses, if done (e.g., sensitivity or subgroup analyses, meta-regression [see Item 16]). | N/A The paper only used legal and policy documents therefore no outcome level assessment was performed. |
| **DISCUSSION** | | |  |
| Summary of evidence | 24 | Summarize the main findings including the strength of evidence for each main outcome; consider their relevance to key groups (e.g., healthcare providers, users, and policy makers). | **Page 30.** “The countries included in this study can be placed in a wide spectrum according to their formulation of drug policy, from Portugal and the UK that have specific legal responses to NPS but have differently focused on harm reduction strategies at one end, to Sweden whose drug-free society goal is not translated into a specific regulation of NPS at the other end. The other EU Member states included in this study may be placed in different points on a continuum, with Poland standing out as the most proliferous in the field of NPS use prevention (see Table 2).”  “In accordance with EU Drugs Strategies implemented since 2000, national drugs policies in the six countries under study may be classified as cross-sectoral as they combine prevention, reduction of drug supply and demand, alongside fight against illicit drug trafficking (see Fig. 3). With respect to NPS specific programmes implemented by most of the countries under study (except for the Czech Republic), it appears that they have focused on the prevention of NPS use, whether through improved information exchange about their chemical composition and hazards or through awareness campaigns on the health risk associated to their use (see Fig 4). |
| Limitations | 25 | Discuss limitations at study and outcome level (e.g., risk of bias), and at review-level (e.g., incomplete retrieval of identified research, reporting bias). | **Page 31.** “The scope of this study only included six European countries, therefore the results cannot be generalized, as more countries should be analysed to get a better picture of the situation across the EU. More research should also be conducted to establish whether strategies towards NPS that are in place have an effect on both the prevention of NPS use and the minimization of the risk associated with their use. Nevertheless, this selection of countries provides a wide overview of the different legal responses and public health strategies adopted in the EU until now, and it may therefore be considered as a benchmark for policy-making process.” |
| Conclusions | 26 | Provide a general interpretation of the results in the context of other evidence, and implications for future research. | **Page 35-36.** “The findings of the study reveal limited development towards harmonisation of national drug policies – particularly with regard to NPS. In the context of the ambiguous position held by the EU in adopting and promoting harm reduction as a prior goal of drug policy, there has been observed a predominance of national approaches to drug use. To tackle the challenge presented by NPS, EU Member states have formulated legislation and public health strategies independently. As a result, national approaches to NPS are in line with their already existing drug policies, reflecting cultural values towards substance abuse and national political interests, while the homogenization at an international level has so far mostly been focused on law enforcement and drug use preventive strategies.” |
| **FUNDING** | | |  |
| Funding | 27 | Describe sources of funding for the systematic review and other support (e.g., supply of data); role of funders for the systematic review. | Page 2: Dr Andres Roman-Urrestarazu’s work received funding from the Gillings Fellowship in Global Public Health, Grant Award YOG054. |

*From:*  Moher D, Liberati A, Tetzlaff J, Altman DG, The PRISMA Group (2009). Preferred Reporting Items for Systematic Reviews and Meta-Analyses: The PRISMA Statement. PLoS Med 6(7): e1000097. doi:10.1371/journal.pmed1000097

For more information, visit: **www.prisma-statement.org**.

Page 2 of 2
